# Community structure and metabolic potentials of the traditional rice beer starter ‘*emao*’

**DOI:** 10.1038/s41598-021-94059-x

**Published:** 2021-07-16

**Authors:** Diganta Narzary, Nitesh Boro, Ashis Borah, Takashi Okubo, Hideto Takami

**Affiliations:** 1 Microbiology and Molecular Systematics Lab, Department of Botany, Gauhati University, Guwahati, Assam India; 2 Yokohama Institute for Earth Sciences, JAMSTEC, Yokohama, 236-0001 Japan; 3 Present Address: Macrogen Japan Corp., 2-4-32 Aomi, Koto-ku, Tokyo, 135-0064 Japan; 4 Present Address: Marine Microbiology, Atmosphere and Ocean Research Institute, The University of Tokyo, 5-1-5 Kashiwanoha, Kashiwa, Chiba 277-8564 Japan

**Keywords:** Genomics, Applied microbiology, Microbial communities, Industrial microbiology

## Abstract

The *emao,* a traditional beer starter used in the North–East regions of India produces a high quality of beer from rice substrates; however, its microbial community structure and functional metabolic modules remain unknown. To address this gap, we have used shot-gun whole-metagenome sequencing technology; accordingly, we have detected several enzymes that are known to catalyze saccharification, lignocellulose degradation, and biofuel production indicating the presence of metabolic functionome in the *emao.* The abundance of eukaryotic microorganisms, specifically the members of Mucoromycota and Ascomycota, dominated over the prokaryotes in the *emao* compared to previous metagenomic studies on such traditional starters where the relative abundance of prokaryotes occurred higher than the eukaryotes. The family Rhizopodaceae (64.5%) and its genus *Rhizopus* (64%) were the most dominant ones, followed by Phaffomycetaceae (11.14%) and its genus *Wickerhamomyces* (10.03%). The family Leuconostocaceae (6.09%) represented by two genera (*Leuconostoc* and *Weissella*) was dominant over the other bacteria, and it was the third-highest in overall relative abundance in the *emao*. The comprehensive microbial species diversity, community structure, and metabolic modules found in the *emao* are of practical value in the formulation of mixed-microbial cultures for biofuel production from plant-based feedstocks.

## Introduction

The ethnic beer (or wine) fermentation process with the use of traditional starter cultures^1^ and without starter cultures (spontaneous)^2^ continues to be prevalent in many parts of the world that possess sociocultural and scientific values. The microorganisms found in the ethnic beer fermentations are diverse^1,2^ and are mostly comprised of yeasts, molds, and bacteria. Each type of ethnic beer fermentation produces desirable end-product(s) with unique taste and flavor due to the complex microbial interactions, succession, and metabolite productions during the fermentation process. A proper understanding of the microbial community structure and the role of individual microorganisms associated with an environmental sample is crucial for bioprospection. However, the key microbial ingredients in the *emao* and the fermentation process in the preparation of several different traditional ethnic beers remain incompletely known.

The analysis of microbial community structure using culture-dependent methods has several limitations, for example, non-cultivability of certain microbes in the laboratory conditions^3^, competition for nutrients, and dominance of some microbes over the others in culture media, and the different growth kinetics among the species. In contrast, culture-independent methods, such as metagenomics through Next Generation Sequencing (NGS), allow the microbial communities to study without the need for microbial isolation and cultivation^3^. Although recently PCR-amplicon sequencing of 16S rRNA genes and ITS rDNA regions have been used for the determination of microbial diversity in many environmental samples including traditional starter cultures, this method is vulnerable to flaws^4,5^ due to the inherent PCR problems including, template competition, primer mismatch, biased amplification due to different template copy number in the analyses of metagenomic DNAs. Alternatively, metagenomic DNAs can be used for whole metagenome sequencing via high throughput NGS platforms, and the data so obtained can be used for protein sequence prediction and subsequent extraction of ribosomal protein (r-protein) sequences for community structure determination of the associated microorganisms. The use of r-protein sequences in taxonomic identification and community structure determination in metagenomics represents a novel methodology in microbial ecology due to the occurrence of a single copy of each ribosomal protein gene in an individual^6^.

**Figure 1:**
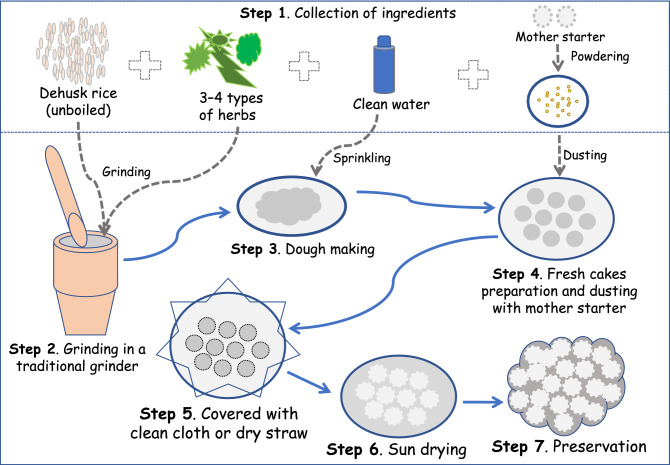
Workflow of the traditional method of *emao* preparation. Rice is used as the base material. The number of herbs might vary as listed in Table 1. Fresh cakes made from mixing rice and herbs are dusted all over with the mother starter powder, thereafter kept covered with a clean cloth or rice straw or fern leaves for 3–4 days. The surface of new cakes turns white due to the growth of microbes. Newly prepared *emao* cakes are sun-dried for 2–3 days and preserved in bags or bamboo containers in aeration condition.

The craft of brewing rice beer (*zu* or *zou*) using *emao* (also written as *amao*) is an age-old tradition among the Bodo (or Boro) community, one of the earliest settlers of the North-East India^7,8^. The origin of the Bodo community and their traditional brewing methodology are not clearly documented. The Bodo, Dimasa, and Garo communities of the North East India might have emerged from a common ancestor about 1500 years ago^9^. There are similarities in their language, culture, and brewing traditions. In the Bodo traditional methodology, the *emao* is prepared from non-sticky rice powder by adding a combination of specific herbs and a small portion of *emao* from the existing stock (Fig. 1, Table 1). The traditional reason for using the herbs in *emao* is to stimulate specific taste and flavor that is unique to Bodo beer, depending on the combination of herbs used. Customarily, 3–5 herbs are used in the preparation of *emao*, sometimes some of these herbs may not be available at the time of its preparation. Several recent studies have reported the microbial diversity occurring on the other similar traditional starters across the world using both culture-dependent^10–15^ and culture-independent methods^16–24^. Nonetheless, the study of microbial diversity in the *emao* is limited, as only four fungal species, one mold and three non-*Saccharomyces* yeasts, have been reported with the use of culture-dependent methods^25–27^. However, bacterial diversity and culture-independent studies as they relate to *emao* have not been reported in the literature.

**Table 1:** List of *emao* samples collected from different Bodo villages of Assam.

S. No	Village address	Local informant	Ingredients used							
			Dehusked rice	Mother starter	*Artocarpus heterophyllus*^a^	*Clerodendrum infortunatum*^b^	*Scoparia dulcis*^b^	*Saccharum officinarum*^a^	*Musa* *balbisiana*^a^	*Ananas comosus*^a^
1	Panichanda, Rani, Kamrup	Deepa Kachari	√	√	√	√	√	–	–	–
2	Saukuchi, Guwahati	Kiran Swargiary	√	√	√	√	√	√		–
3	Boragari, Dotoma, Kokrajhar	Bhaigo Brahma	√	√	√	√	√	√	√	–
4	Goybari, Gossaigaon, Kokrajhar	Sambashi Brahma	√	√	√	√	√	–	√	–
5	Tengapara, Kokrajhar	Reena Narzary	√	√	√	√	√	–	–	–
6	Lechera, Baksa	Laishri Narzary	√	√	√	√	√	–	–	√

Tick (√) mark represents present and dash (–) mark represents the absence of the ingredient. The use of *Artocarpus heterophyllus* and *Clerodendrum infortunatum* along with rice powder and mother starter is common, whereas the other herbs are used alternatively depending on the availability of the plants at the time of *emao* preparation.

^a^Leaves are used.

^b^Leaves or twigs containing leaves and flowers are used.

The occurrence of amylolytic, alcohol-producing and lactic acid bacteria (LAB) that represent three major groups of microorganisms, in several ethnic beer starters has been reported^1^. In addition, the occurrence of acetic acid bacteria such as *Acetobacter orientalis* and *A. pasteurianus* in traditional starter *Banh men*^28^, and *Gluconobacter* sp. in traditional starter *Marcha*^29^ were also reported. Thus, in most traditional beer fermentation processes several microorganisms work together in consortia mode, which produces unique beer. The traditional method entails the preparation of starter culture and maintenance; however, the substrate used for fermentation varies from one locality to the next, and one community to the other^1^. These variations also add diversity to the microbial species that catalyze the production of beer, therefore the fermented products are expected to taste different from one to the other. Close to two dozens of traditional beer starters are found throughout the North East India^30^, and only a few of them have been studied using culture-independent methods^16,22,24^. As biofuel-producing microorganisms are important for sustainable biowaste management and energy production, therefore, the identification of biofuel-producing microorganisms is an ongoing effort. The production and use of biofuels derived from organic materials including biowastes are necessary, thereby reducing the CO_2_ emissions associated with the fossil fuel combustion. The traditional beer starter cultures of Asian origin are already in use for bio-ethanol production from cereals or fruits. Therefore, these starter cultures could potentially serve as a source of other forms of biofuel-producing microorganisms too. Here, for the first time, we report the microbial community structure in the *emao* based on the ribosomal protein sequences derived from the whole-metagenome sequences and profile the metabolic potentials of *emao* in relation to biofuel production.

## Results

### Whole-metagenome overview

The number of nucleotide sequence reads recorded in the *emao* sample was 13,060,410, which gave a total of 6,530,205 merged reads with ≥ 400 nucleotides per sequence (Table 2). We obtained a total of 1,285,880 amino acid (AA) sequences having ≥ 50 AA per sequence, which was subsequently used as an input for taxonomic binning, enzyme identification, and metabolic module analyses.

**Table 2:** Basic sequence information on *emao* metagenome data.

Total sequence reads (nt in pairs)	Total number of merged reads (not less than 400 nt per contig)	Total number of AA sequences (not less than 50 aa per sequence)	AA sequences annotated as ribosomal proteins in Genomaple for all organisms (M91000)	AA sequences annotated as CAZymes after homology search against dbCAN	AA sequences annotated as BPZymes after homology search against BioFuelDB
13,060,410	6,530,205	1,285,880	8562	19,702	34,493

The nucleotide sequences were obtained after shot-gun sequencing of the six-pooled sample of *emao* in Illumina HiSeq 2500 Platform.

*CAZymes* carbohydrate-active enzymes, *BPZymes* biofuel producing enzymes.

### Community structure and species diversity

**Figure 2:**
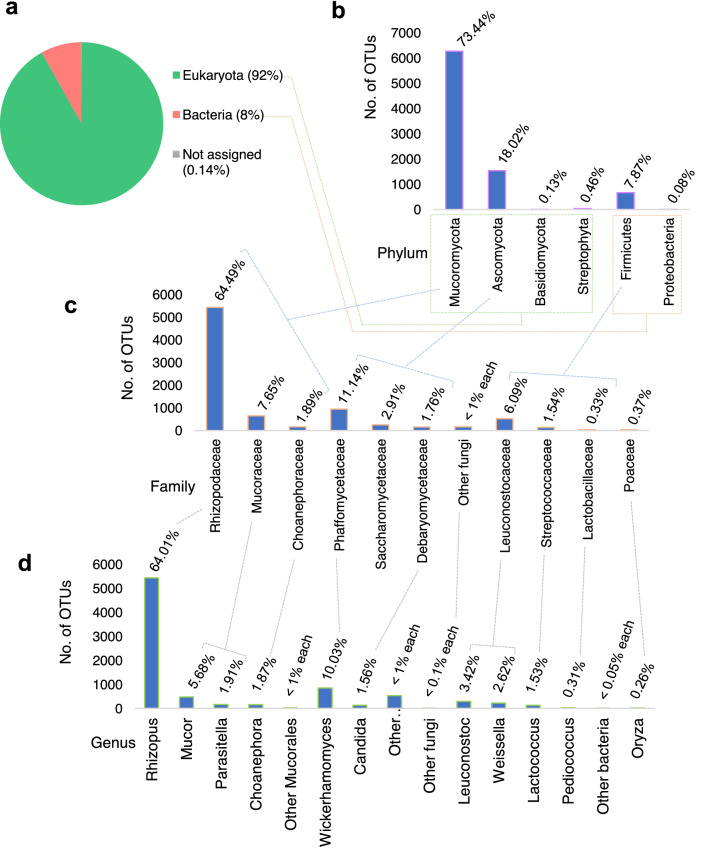
Estimation of community structure in the *emao* based on ribosomal protein sequence analysis. The ratio of molds, yeasts, and bacteria was 9:2:1. (**a)** At domain level; (**b)** at phylum level; (**c)** at family level; (**d)** at genus level.

We retrieved a total of 8562 non-redundant ribosomal protein (r-protein) sequences from the module M91000 for all organisms (Table 2) and the taxonomic binning of these r-protein sequences revealed 92% Eukarya and 8% Bacteria with a ratio of 9:2:1 for Mucoromycota (molds), Ascomycota (yeasts), and Firmicutes (bacteria), respectively (Fig. 2a). The relative abundance of molds was the highest (73.44%), followed by yeasts (18.02%) and lactic acid bacteria (LAB) (7.87%) in *emao* (Fig. 2b). However, the yeast species diversity was four times higher than the bacteria and molds in *emao* (Supplementary Data Table S1). The relative abundance of the genus *Rhizopus* and its family Rhizopodaceae were the highest among the groups in *emao* (Fig. 2c,d). Among the yeasts, the relative abundance of the genus *Wickerhamomyces* and its family Phaffomycetaceae were the highest.

**Figure 3:**
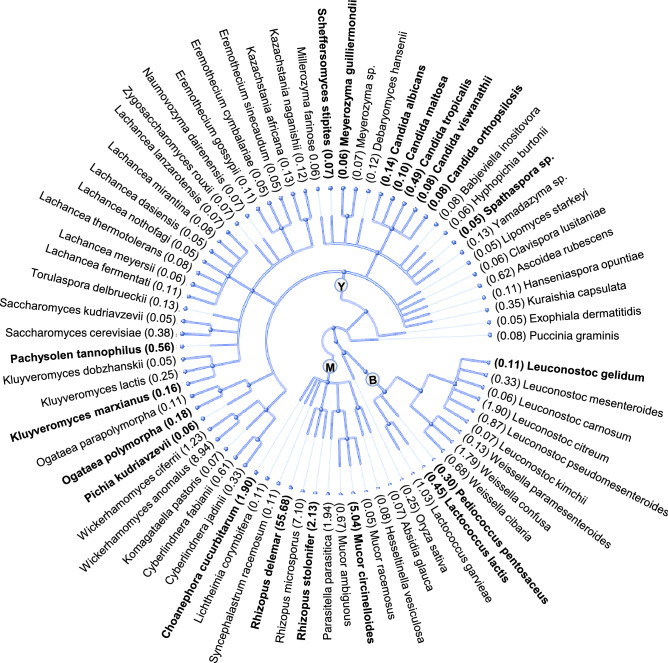
Phylogenetic tree of species as recorded in the *emao.* A total of 74 microbial species and 1 rice species were identified based on a ribosomal protein sequence homology search against the NCBI-nr database. The values within parentheses indicate the relative abundance in percent. Species names in bold (total 19) indicate the lignocellulose/pentose metabolizer as listed in Supplementary Data Table S3). *B* bacteria, *M* molds, *Y* yeasts.

We identified a total of 74 microbial species in *emao* based on r-protein sequences (Fig. 3, Supplementary Data Table S1). *Rhizopus delemar* (syn. *R. oryzae*) was the highest with 56% overall relative abundance (ORA), followed by *R. microsporus* (7% ORA) and *Mucor circinelloides* (5% ORA) among the molds. Among the yeasts, *Wickerhamomyces anomalus* was the most dominant (9% ORA), followed by *W. ciferrii* (1% ORA), *Ascoidea rubescens* (0.6% ORA), *Cyberlindnera fabiani* (0.6%), *Pachysolen tannophilus* (0.6% ORA), *Candida tropicalis* (0.5% ORA), *Saccharomyces cerevisiae* (0.4% ORA), and a few more ethanol-producing species with low ORA (Supplementary Data Table S1). Among LAB, the most dominant species was *Leuconostoc mesenteroides* (1.9% ORA), followed by *Weissella confusa* (1.8% ORA) and *Lactococcus garvieae* (1% ORA).

The microbial diversity compared to other traditional beer starters revealed that the *emao* exhibits some bacterial species, *Pediococcus pentosaceus*, *Leuconostoc citreum*, *L. mesenteroides*, *L. pseudomesenteroides*, *Weissella ciberia*, *W. confusa*, *W. paramesenteroides*, *Lactococcus lactis*, *L. gravieae*; and yeast species, *Candida tropicalis*, *Debariomyces hansenii*, *Hypopichia burtonii*, *Pichia kudriavzevii*, *Meyerozyma guilliermondii*, *Millerozyma farinosa*, *Clavispora lusitaniae*, *Wickerhamomyces anomalus*, *W. ciferrii*, *Ogataea parapolymorpha*, *Kluveromyces dobzhanskii*, *K. lactis*, *Torulaspora delbrueckii*, *Saccharomyces cerevisiae*; and mold species, *Lichthieimia corymbifera*, *Mucor circinelloides*, *M. racemosus*, *Rhizopus delemar*, *R. microsporus*, *R. stolonifer* share commonality to other traditional starter cultures, *Nuruk* from Korea^21,31,32^, *Marcha*^29^ and *Xaj-pitha*^22^ from India, *Daku* from China^11,19^, and *Koji* from Japan^22^ (Supplementary Data Table S1). Some of the dominant species recorded in *emao* were also common in several other traditional beer starters considered for comparison. Of 24 other traditional starters (OTS)^10–47^ compared to *emao*, the dominant microbial species, *W. anomalus* was found common in 20 OTS, *S. cerevisiae* in 15 OTS, *R. delemar* in 13 OTS, *M. circinelloides* in 12 OTS, *Pediococcus pentosaceus* in 9 OTS, *R. microsporus* in 7 OTS, and *Torulaspora delbrueckii*, *L. mesenteroides* and *Candida tropicalis* in 6 OTS (Supplementary Data Table S1). However, the other dominant species, *R. stolonifer*, *Choanephora cucurbitarum*, *Parasitella parasitica*, *W. ciferrii*, *C. fabianii*, *A. rubescens*, *L. citreum*, *W. confusa*, and *L. garvieae* recorded in *emao*, were found only in a few OTS. The rest of the microbial species which accounted for 51% of the total species detected in *emao* were not reported previously in the 24 OTS considered for comparison (Supplementary Data Table S1).

### Identification of carbohydrate-active enzymes (CAZymes) and biofuel producing enzymes (BPZymes) in *emao*

**Figure 4:**
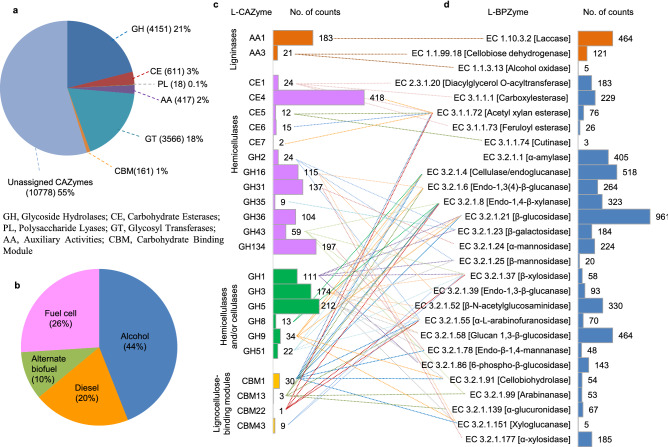
Lignocellulolytic enzymes predicted in *emao* based on carbohydrate-active enzymes (CAZymes) and biofuel producing enzymes (BPZymes) analyses. A complete list of CAZymes and BPZymes as recorded in *emao* is given in Supplementary Data Fig. S1 & S2, respectively. (**a)** Pie chart indicating the percentage of CAZyme families assigned by Hotpep. The value within parentheses is the total count. (**b)** Relative percentage of BPZyme categories as classified in BioFuelDB. (**c)** Lignocellulolytic CAZyme sub-families segregated activity-wise. (**d)** Lignocellulolytic BPZymes with Enzyme Commission (EC) number extracted from (**b)** and correlated to (**c)** (dotted lines).

A total of 19,702 CAZymes^48^ and 34,493 BPZymes^49^ were recorded in *emao* (Fig. 4a,b, Supplementary Data Fig. S1 & S2). Hotpep^50^, a CAZyme assigning program successfully assigned only 45% of the total CAZymes detected in *emao* into six CAZyme families and 123 CAZyme sub-families. Among the CAZyme families, glycoside hydrolases (GH) were recorded as the most prevalent (21%), followed by glycosyltransferases (GT, 18%), whereas the rest four types of CAZymes were comparatively less prevalent (≤ 3%). We also emphasized identifying the lignocellulolytic enzymes occurring in *emao* as essential for mobilizing lignocellulosic substrates into useful products such as biofuel. As such, there is no database for lignocellulolytic enzymes; therefore, we compared the CAZymes and BPZymes of *emao* to the previous reports on lignocellulolytic enzymes^51,52^ and identified a total of 1929 lignocellulolytic CAZymes and 5576 lignocellulolytic BPZymes in *emao* (Fig. 4c,d). A comparison of lignocellulolytic enzymes of *emao* to pill bug (*Armadillidium vulgare*) gut microbiome^52^ revealed seven times higher lignin modifying enzymes, four times higher hemicellulases, and four times higher hemicellulases and/or cellulases in *emao*, in contrast to four times higher lignocellulose-binding modules in pill bug. In BPZyme analysis, enzymes associated with alcohol production were found two times higher than the enzymes associated with diesel production and fuel cells. We could identify 44% ethanol-producing, 26% fuel-cell producing, 20% diesel producing, and 10% alternate-biofuel producing enzymes (Fig. 4b).

### Metabolic and physiological potentials in *emao*

**Figure 5:**
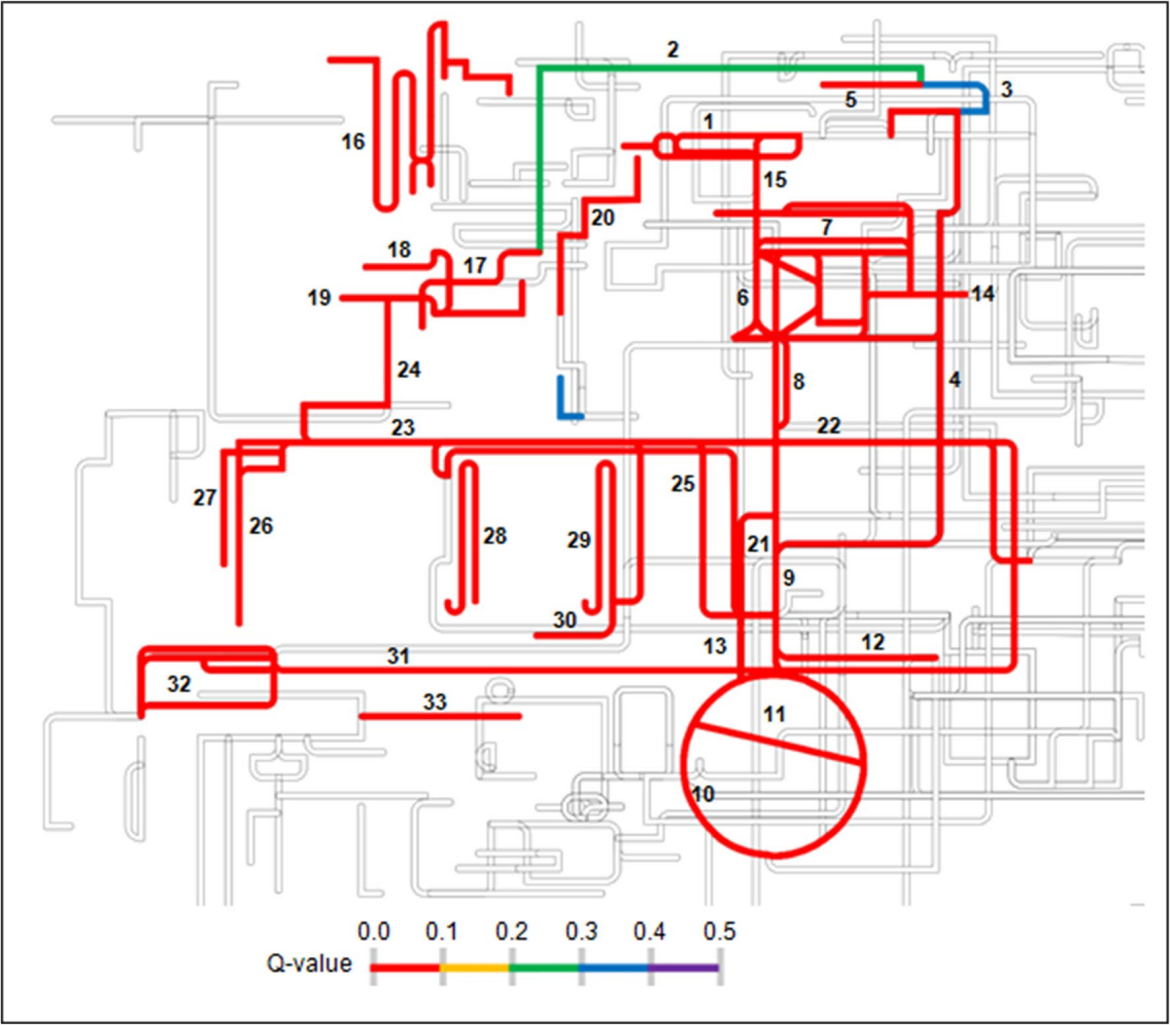
Carbohydrate and lipid metabolic maps in the *emao*. Metabolic modules with a Q-value less than 0.5 for whole microbial communities (WC) were shown in the map (Q-values are provided in Supplementary Data Table S2). The probability of occurrence of a metabolic module increases with decreasing Q-value as the latter is zero if all the genes necessary for a module is complete^55^. This coarse-grained map was created using the KEGG Atlas map^56,87^. 1, Galactose degradation; 2, D-galacturonate degradation (fungi); 3, D-galacturonate degradation (bacteria); 4, D-glucuronate degradation; 5, Pectin degradation; 6, Embden-Meyerhof Pathway (glycolysis); 7, Pentose phosphate pathway; 8, Glycolysis core module; 9, Pyruvate oxidation; 10, Citrate cycle; 11, Glyoxylate cycle; 12, Melanoate semialdehyde pathway; 13, Gluconeogenesis; 14, PRPP biosynthesis; 15, Nucleotide sugar biosynthesis; 16, N-glycan metabolism; 17, Acyl glycerol degradation; 18, Phosphatidylcholine biosynthesis; 19, Phosphatidylethanolamine biosynthesis; 20, GPI-anchor biosynthesis, core oligosaccharide; 21, Inositol phosphate metabolism; 22, Ceramide biosynthesis; 23, Sphingosine biosynthesis; 24, Sphingosine degradation; 25, Fatty acid biosynthesis, initiation; 26, Fatty acid biosynthesis, elongation; 27, Fatty acid biosynthesis, elongation (ER); 28, Fatty acid biosynthesis, elongation (mitochondria); 29, Beta-oxidation; 30, Beta-oxidation acyl-CoA synthesis; 31, C5 isoprenoid biosynthesis (Mevalonate); 32, C10-20 isoprenoid biosynthesis; 33, Ergocalciferol biosynthesis.

An analysis of amino acid sequences in Genomaple^53,54^ revealed the most feasible functional modules with significant module completion ratio (MCR) and *Q*-values at an individual taxonomic rank (ITR) or whole microbial count (WC) level. Any module having 100% MCR and/or less than 0.5 *Q*-value is considered significant and feasible^53,55^. A total of 489 metabolic KEGG (Kyoto Encyclopedia of Genes and Genomes) modules with > 0% MCR (WC) scores were recorded in *emao*, out of which 46% (*i.e.*, 28% out of total 804 existing modules in KEGG^56^) had 100% MCR (WC), and that could be the crucial modules in determining the functionality and uniqueness of *emao* in beer fermentations (Supplementary Data Table S2). The carbohydrate and lipid metabolisms are involved directly or indirectly in plant biomass degradation and biofuel production. Therefore, we focused on carbohydrate and lipid metabolic modules having significant MCR and *Q*-values (Fig. 5, Table 3).

**Table 3:** Carbohydrate and lipid metabolism pathways as predicted in *emao* by MAPLE system.

Sl. No	Module ID	Pathway name	MCR % (ITR)	MCR % (WC)	Q-value (WC)
**Central carbohydrate metabolism**					
1	M00001	Glycolysis (Embden-Meyerhof pathway), glucose = > pyruvate	100	100	0
2	M00002	Glycolysis, core module involving three-carbon compounds	100	100	0
3	M00003	Gluconeogenesis, oxaloacetate = > fructose-6P	100	100	0
4	M00307	Pyruvate oxidation, pyruvate = > acetyl-CoA	100	100	0
5	M00009	Citrate cycle (TCA cycle, Krebs cycle)	87.5	87.5	0
6	M00010	Citrate cycle, first carbon oxidation, oxaloacetate = > 2-oxoglutarate	100	100	0
7	M00011	Citrate cycle, second carbon oxidation, 2-oxoglutarate = > oxaloacetate	80	80	0
8	M00004	Pentose phosphate pathway (Pentose phosphate cycle)	100	100	0
9	M00006	Pentose phosphate pathway, oxidative phase, glucose 6P = > ribulose 5P	100	100	0
10	M00007	Pentose phosphate pathway, non-oxidative phase, fructose 6P = > ribose 5P	100	100	0
11	M00580	Pentose phosphate pathway, archaea, fructose 6P = > ribose 5P	100	100	0
12	M00005	PRPP biosynthesis, ribose 5P = > PRPP	100	100	0
13	M00008	Entner-Doudoroff pathway, glucose-6P = > glyceraldehyde-3P + pyruvate	75	75	0.5
14	M00308	Semi-phosphorylative Entner-Doudoroff pathway, gluconate = > glycerate-3P	80	80	0.875
**Other carbohydrate metabolism**					
15	M00012	Glyoxylate cycle	100	100	0
16	M00013	Malonate semialdehyde pathway, propanoyl-CoA = > acetyl-CoA	100	100	0
17	M00632	Galactose degradation, Leloir pathway, galactose = > alpha-D-glucose-1P	100	100	0
18	M00014	Glucuronate pathway (uronate pathway)	62.5	75	0.751
19	M00630	D-Galacturonate degradation (fungi), D-galacturonate = > glycerol	75	75	0.276
20	M00631	D-Galacturonate degradation (bacteria), D-galacturonate = > pyruvate + D-glyceraldehyde 3P	60	80	0.376
21	M00061	D-Glucuronate degradation, D-glucuronate = > pyruvate + D-glyceraldehyde 3P	100	100	0
22	M00081	Pectin degradation	100	100	0
23	M00549	Nucleotide sugar biosynthesis, glucose = > UDP-glucose	100	100	0
24	M00554	Nucleotide sugar biosynthesis, galactose = > UDP-galactose	100	100	0
25	M00793	dTDP-L-rhamnose biosynthesis	100	100	0
**Fatty acid metabolism**					
26	M00082	Fatty acid biosynthesis, initiation	100	100	0
27	M00083	Fatty acid biosynthesis, elongation	100	100	0
28	M00085	Fatty acid biosynthesis, elongation, mitochondria	100	100	0
29	M00415	Fatty acid biosynthesis, elongation, endoplasmic reticulum	100	100	0
30	M00086	beta-Oxidation, acyl-CoA synthesis	100	100	0
31	M00087	beta-Oxidation	100	100	0
**Lipid metabolism**					
32	M00089	Triacylglycerol biosynthesis	75	75	0.956
33	M00098	Acylglycerol degradation	100	100	0
34	M00090	Phosphatidylcholine (PC) biosynthesis, choline = > PC	100	100	0
35	M00091	Phosphatidylcholine (PC) biosynthesis, PE = > PC	100	100	0
36	M00092	Phosphatidylethanolamine (PE) biosynthesis, ethanolamine = > PE	100	100	0
37	M00093	Phosphatidylethanolamine (PE) biosynthesis, PA = > PS = > PE	100	100	0
38	M00130	Inositol phosphate metabolism, PI = > PIP2 = > Ins (1,4,5) P3 = > Ins (1,3,4,5) P4	75	75	0.739
39	M00094	Ceramide biosynthesis	100	100	0
40	M00099	Sphingosine biosynthesis	100	100	0
41	M00100	Sphingosine degradation	100	100	0
**Glycan metabolism**					
42	M00055	N-glycan precursor biosynthesis	100	100	0
43	M00072	N-glycosylation by oligosaccharyltransferase	100	100	0
44	M00073	N-glycan precursor trimming	100	100	0
45	M00074	N-glycan biosynthesis, high-mannose type	100	100	0
46	M00065	GPI-anchor biosynthesis, core oligosaccharide	100	100	0
**Sub-category: terpenoid backbone biosynthesis**					
47	M00095	C5 isoprenoid biosynthesis, mevalonate pathway	100	100	0
48	M00849	C5 isoprenoid biosynthesis, mevalonate pathway, archaea	100	100	0
49	M00364	C10-C20 isoprenoid biosynthesis, bacteria	100	100	0
50	M00366	C10-C20 isoprenoid biosynthesis, plants	50	100	0
51	M00367	C10-C20 isoprenoid biosynthesis, non-plant eukaryotes	100	100	0
**Sterol biosynthesis**					
52	M00101	Cholesterol biosynthesis, squalene 2,3-epoxide = > cholesterol	80	80	0.751
53	M00102	Ergocalciferol biosynthesis	100	100	0
54	M00109	C21-Steroid hormone biosynthesis, progesterone = > cortisol/cortisone	50	75	0.574

Metabolic pathways with 75% or more module completion ratio (MCR) were shown for the whole microbial community (WC).

*ITR* individual taxonomic rank.

The presence of both 100% MCR for the Embden-Meyerhof pathway with zero *Q*-value and 75–80% MCR (0.5–0.85 *Q*-value) for the Entner-Doudoroff pathway (which occurs mainly in some bacteria), revealed the presence of feasible alternative glycolytic pathways in *emao* (Table 3). Besides, 100% MCR with zero *Q*-value for pectin, galactose, d-galacturonate, and d-glucuronate degradation pathways are noteworthy, which signifies the possibility of metabolizing those substrates by the microorganisms associated with *emao* (Fig. 3). Acyl-CoA is necessary for the synthesis of fatty acids precursor, acetyl-CoA^57,58^. Thus, 100% MCR for both the beta-oxidation module and acyl-CoA synthesis module suffices the fatty acids and isoprenoid biosynthesis potentiality in *emao*. The presence of 100% MCR for mitochondrial and endoplasmic fatty acid biosynthesis modules and 100% MCR for lipid biosynthesis modules corroborate the involvement of eukaryotes (mainly fungi) in biodiesel production.

## Discussion

The analyses of yeasts, molds and LAB found in the *emao* showed the presence of major groups of microbial compositions similar to the Asian ethnic beer starters including *Nuruk*, *Marcha*, *Thiat*, *Hamei*, *Humao*, and *Xaj-pitha* (Supplementary Data Table S1)*.* However, a certain degree of variations is evident among the ethnic starters in their microbial compositions at the species or genus level. One could surmise that variations could be due to the ingredient used and preparation methods followed by different communities. The *emao* preparation entails rice as a base material and the variation often occurs in the use of quantity and number of herbal species. Of 7–8 different types of herbs, the use of at least 2–3 is the traditional norms of the Bodo people in *emao* preparation, although the herbs are often used alternatively depending on the availability of the herbs at the time of *emao* preparation [Source: Local informants]. *Emao* starters usually produce rice beer containing a maximum of about 17% (w/v) ethanol^59^, however, the ethanol concentrations can vary depending on the duration of the fermentation period. We found a typical community structure in *emao* using ribosomal protein-based taxonomic binning where the yeasts, molds, and LAB were seen at a ratio of 9:2:1, respectively. We consider this community structure of *emao* might be appropriate for producing traditional *zou* or *joubishi* containing 5–17% (w/v) ethanol^59–61^ that might have had garnered thousand years ago and is still being maintained in its pristine form by the Bodo peoples through their traditional practices. The findings of higher relative abundance of Firmicutes than Proteobacteria, and higher fungal species counts than bacteria in *emao* (Fig. 5) differ from the community structures as reported previously in the traditional beer starters *Xaj-pitha*^22^, *Marcha* and *Thiat*^29^ from the region. Such a deviation is likely due to differences in methods and approaches followed.

The yeasts, molds, and LAB play unique roles in alcoholic beverage fermentation, especially when starch is the feedstock. Molds are mostly aerobic, do the saccharification, and sensitive to ethanol except for a few species that can produce a low level of ethanol^62^. The presence of a high number of saccharifying and/or lignocellulose degrading microorganisms is always advantageous and desirable at the beginning of fermentation while using plant-based substrates. Some non-*Saccharomyces* yeast does both saccharification and ethanol production simultaneously but is mostly non-tolerant to high ethanol concentrations^63–66^. Ethanol-sensitive microbes are subsequently killed or arrested by increasing ethanol concentrations at the later stages of ethanol fermentation^67^. Ethanol production and tolerance level of *Saccharomyces* yeasts also vary from strain to strain, although they are generally high ethanol tolerant^68–70^. Wild *S. cerevisiae* cannot utilize starch or other complex carbohydrates directly due to the lack of degrading enzymes for those substrates^71^ for which they are dependent on other saccharifying microbes.

A low proportion of LAB is desirable in beer fermentation as they can perform some necessary functions despite being known as spoilage agents^72,73^. They mainly produce lactic acid besides producing bacteriocins against some human pathogens, and they are responsible for maintaining low pH, keeping quality, and taste enhancement in beer^74–77^. We did not find any acetic acid-producing bacteria (*Acetobacter* spp.) in *emao,* which is a good indicator for this traditional starter of the Bodo community. Acetic acid bacteria are responsible for beer defects^78,79^, which often happens due to contamination of such spoilage agents if hygienic conditions are compromised during the preparation of starter culture or beer fermentation.

Our findings of the saccharifying, lignocellulolytic (Supplementary Data Table S3), and different biofuels producing microorganisms in corroboration with probable metabolic functionomes (Fig. 5, Table 3) in *emao* is noteworthy, and the current community structure as found in *emao* could help develop an effective lignocellulolytic bio-consortia necessary for second-generation biofuel production. Many simple carbohydrate degrading microorganisms do not easily break down the pentose sugars such as D-xylose and L-arabinose that comprise up to 20% of lignocellulosic biomass^80^. The breaking of lignocellulose into its subunits and subsequently mobilizing them as energy sources for biofuel production is complex. It requires various enzymes to catalyze the metabolic processes, and an organism bearing all the essential enzymes together is rare. However, further experimentation is required to validate the utility of *emao* as such or in combination with other lignocellulolytic microbes. As the species diversity and the metabolic potentials of *emao* as reflected from this study are diverse, a pyramiding of target microorganisms from such a natural bio-consortium towards achieving a target product from specific substrates by necessary functional potentials could be a new avenue in tapping natural bioresources for bioprospection. Such an alternative approach can pave the way for bio-consortia formulation to produce biofuels from lignocellulosic materials.

The Genomaple system provides an effective platform to visualize the module completion ratio (MCR) along with the taxonomic information at Phylum or Class level that reflects their functional activity in completing different metabolic modules in any environmental sample considered for an investigation^53–55,81^. However, it is dependent on the KEGG database, which includes only the species with complete genome sequence information. Draft genome information of mold species belonging to Zygomycota is available in the public domain, but not the complete genome information due to which none of these are considered yet in the KEGG database^56^. In absence of reference taxonomic information, it becomes difficult to specify and segregate the predicted metabolic modules among the taxonomic groups below Phylum or Class level. For the same reason, we were unable to specify and segregate the metabolic modules among the molds and yeasts in this functional metagenome study on *emao*. Molds are ubiquitous in distribution and play critical ecological roles similar to other fungal groups^82^. Complete genome information on molds is needed in the public domain to understand better their roles in natural environments and brewing.

## Conclusions

The present study is the first to characterize the comprehensive community structure and probable metabolic potentials of the microorganisms associated with the traditional starter culture *emao*. The presence of diverse groups of microorganisms in corroboration with amylolytic, lignocellulolytic, biofuels producing enzymes as recorded in *emao* is noteworthy. It could be a pathfinder in the field of microbial consortia bioformulation for biofuel production from otherwise recalcitrant plant biomasses. We found the ribosomal protein-based community structure enumeration a suitable approach in metagenome study. Complete genome information on molds is equally essential in the line of other fungal groups to better understand their roles in traditional brewing and other natural environments. Some dominant microbial species found in *emao* are similar to some other traditional starters reported previously from the region. Therefore, a comparative study of traditional beer starters that carry microbial genetic information could be of paramount significance in the elucidation and understanding of the history of human population migration and civilization through the ages, like archaeology and philology.

## Materials and methods

### Sample collection

**Figure 6:**
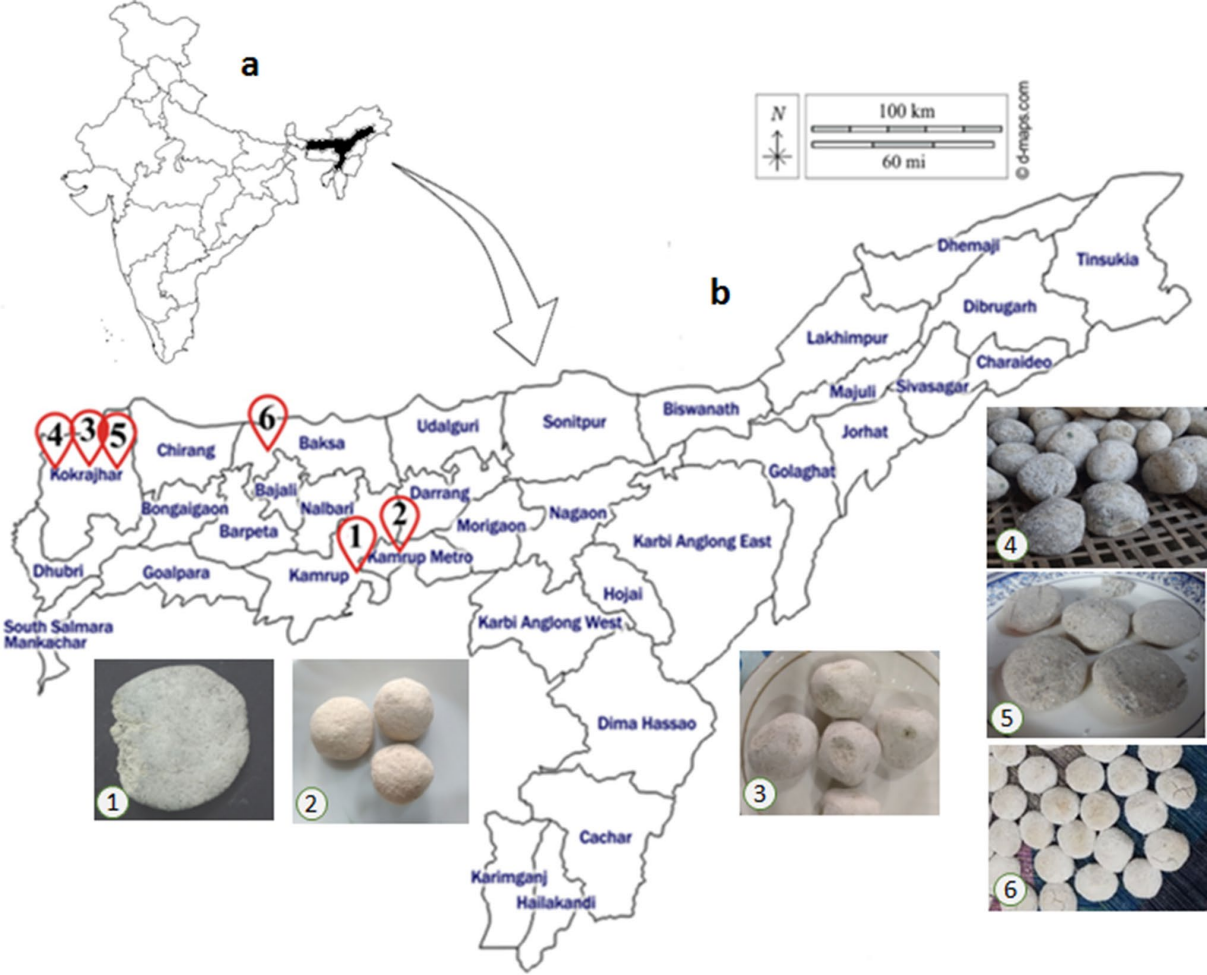
Map showing the *emao* sampling sites. **(a)** Map of India with Assam state highlighted in dark, **(b)** map of Assam marked with the *emao* sampling sites (red). Sample numbers (S. No.) are as given in Table 1. The map templates were taken from d-maps.com (https://d-maps.com/pays.php?num_pay=84&lang=en & ) and the sampling sites were marked upon it using Adobe Photoshop CS (Version 8.0, 2003). The photographs of the *emao* samples were taken by D. Narzary and Nitesh Boro.

Six representative *emao* samples traditionally prepared by the Bodo people were collected from different Bodo-dominated villages of Assam (Table 1, Fig. 6). Information on the method of preparation and herbs used in starter culture were also recorded. We ensured that no beer defect had been experienced during sample collection in using *emao* from the same stock that we brought for scientific investigation. Traditionally, more than one-year-old *emao* samples are generally not used for rice beer fermentation; instead, the starter culture is revived in the fresh rice-based medium before completing one year. Therefore, only the active samples, i.e., less than one year from the date of preparation, were considered for this study.

### Total DNA isolation

Two grams of each of six *emao* samples were taken and mixed by grinding in pre-sterilized mortar by a pestle, from which a 10 g sample was taken to isolate the total DNA. To recover quality metagenomic DNA, we modified the method of Zhou et al.^83^ where the extraction buffer was supplemented with 1% activated charcoal and 10 mM MgCl_2_ as recommended by Sharma et al.^84^, and the DNA obtained from the modified method of Zhou et al.^83^ was again purified with the MoBio DNA isolation kit (QIAGEN, Cat. No. 12888–100). The steps we followed are described below. Liquid nitrogen was used for effective sample grinding and the sample was transferred into 18 ml of extraction buffer [100 mM Tris–HCl (pH 8.0), 100 mM sodium EDTA (pH 8.0), 100 mM sodium phosphate (pH 8.0), 1.5 M NaCl, 1% CTAB, 10 mM MgCl_2_ and 1% activated charcoal] before thawing. A volume of 100 μl proteinase K (10 mg/ml) was added to the tube, mixed properly by vortexing, and incubated at 37 °C for 30 min in a water bath with gentle end-over-end inversions every 5 min. After that, 2 ml of 20% SDS was added, mixed by vortexing, and incubated at 65 °C for 2 h with gentle end-over-end inversions every 30 min. The sample was allowed to cool up to room temperature (RT), and the supernatant was collected in a fresh centrifuge tube after centrifugation at 6000×*g* for 10 min at RT. The supernatant was mixed with an equal volume of chloroform: isoamyl alcohol (24:1, vol/vol) by inverting the tube gently. The aqueous phase was recovered by centrifugation at 10,000×*g* for 10 min at RT and then precipitated with 0.6 volume of pre-chilled isopropanol at RT for one h. The pellet of crude nucleic acids was obtained by centrifugation at 12,000×*g* for 20 min at RT, washed with chilled 70% ethanol, and resuspended in sterile deionized water to make the final volume 700 μl. The DNA solution so obtained was purified with a MoBio DNA isolation kit, and the steps from the treatment of C4 solution onwards were followed according to the procedure of the kit. DNA was stored at –20 °C till sending for whole-metagenome sequencing to the service provider.

### Whole-metagenome sequencing

The whole-genome sequencing of our metagenomic DNA was outsourced to the AgriGenome Labs Pvt. Ltd., Kerala, India, and the sequencing was done using the Illumina HiSeq 2500 Platform. The quality of the DNA was confirmed in Qubit Fluorometer and agarose gel electrophoresis before the library preparation. The Genomic DNA was fragmented using Covaris M220 for 500 bp, and the library was prepared using NEBNext Ultra DNA Library Prep Kit. The library quality was checked using Agilent Tapestation 2200. The quantity was estimated using Qubit 2.0. The libraries were sequenced in the HiSeq 2500 platform for 2 × 250 bp read length generating the required data. The FASTQ files generated by the Illumina HiSeq platform were trimmed with Cutadapt (Version 1.8.1)^85^ to remove the adapters.

### Sequence assembly, annotation, and evaluation of potential metabolic modules

The forward and reverse DNA sequences in FASTQ format were submitted to the MAPLE Submission Data Maker (MSDM) pipeline for quality filtering, forward and reverse sequence assembly, and translation to amino acid sequences where the nucleotide sequences with a minimum base quality score of Q20, minimum 80% of quality bases in each sequence, and minimum 400 bp in each merged read were set to get high-quality amino acid (AA) sequences with a minimum cut-off length of 50 AA in FASTA format^55^. Genomaple ver. 2.3.2 (formerly MAPLE) is a powerful tool that can predict the metabolic functionomes from nucleotide or amino acid sequences as detailed in MAPLE references^53,54,81,86^. The AA sequence file so obtained was then analyzed in Genomaple ver. 2.3.2 (formerly MAPLE) server opting for the GHOST X search engine with the single-direction best hit annotation for all organisms in KEGG. The module completion ratio (MCR) and *Q*-value at the individual taxonomic rank (ITR) and the whole microbial community (WC) level were retrieved from MAPLE results, and the KEGG Orthology (KO) genes assigned by Genomaple were used for subsequent taxonomic binning, CAZyme and BPZyme analyses. The module information generated by MAPLE was used to create the coarse-grained metabolic maps of KEGG modules using the KEGG Atlas map^87^ as a reference. The metabolic map for carbohydrate and lipid metabolism was created separately for the modules having *Q-*values^53^ lower than 0.5 to understand the biomass degradation and biofuel production potentiality in *emao*.

### Taxonomic binning

The KOs assigned to the ribosomal protein module for all organisms (M91000) was extracted back from the MAPLE input file using NCBI-blast dbcmd command, which was then subjected to homology search against the non-redundant NCBI-nr protein database to assign the taxonomic identity for each sequence in GHOSTX program^88^ using the top hit option. GHOSTX result was manually curated to parse the species name against each sequence, which was then meganized and visualized in MEGAN^89^ Community Version (V6.12.5). The identified species names were uploaded to the NCBI Tree Viewer (https://www.ncbi.nlm.nih.gov/projects/treeview/) to generate the circular phylogenetic tree.

### CAZyme identification

The AA sequences created by the MSDM pipeline (version 1.0)^55^ were used as query files in HMMR hmmscan program (version 3.2.1)^90^ against dbCAN database^91^ as a reference with an E-value threshold of 1e-5 to predict CAZymes. The AA sequences detected as CAZymes^48^ were retrieved back from the input file, and the duplicates were removed using some basic perl and shell commands to get the non-redundant FASTA sequences. Then the non-redundant sequences so obtained were assigned to different CAZyme categories using the Hotpep program^50^.

### BPZyme identification

The AA sequences generated by MSDM in FASTA format were used to identify the enzymes involved in biofuel production as a query file in the HMMR phmmer program^90^ against BioFuelDB^49^ as a reference with an E-value threshold of 1e−5. The sequence homology and the corresponding EC number of non-redundant AA sequences identified as the biofuel-producing enzymes were reconfirmed using the GHOSTX homology search (top hit only) against the BioFuelDB. All the ECs were then segregated into different biofuel categories as classified by Chaudhary et al.^49^, and the total enzyme counts for each category were obtained.

## Supplementary Information

Supplementary Figure S1.

Supplementary Figure S2.

Supplementary Table S1.

Supplementary Table S2.

Supplementary Table S3.

## Data Availability

The nucleotide sequence raw data (DNS_R1 and DNS_R2) were submitted to the European Nucleotide Archive (ENA) and the study accession number is PRJEB45760. The amino acid sequence data generated by the MAPLE Submission Data Maker (DNS1), MAPLE assigned KOs (DNS2), KEGG metabolic modules (DNS3), and ribosomal protein-based community structure information in RMA format (DNS4) are available through figshare (https://doi.org/10.6084/m9.figshare.8868689). Any other relevant data are available from the corresponding author upon reasonable request.
