# Supplementary material for: Community structure and metabolic potentials of the traditional rice beer starter ‘emao’
Source: Sci Rep. 2021 Jul 16;11:14628. doi: 10.1038/s41598-021-94059-x (PMC8285430; doi:10.1038/s41598-021-94059-x)

Community structure and metabolic potentials of the traditional rice beer starter ‘*emao*’

Diganta Narzary[ab*], Nitesh Boro[a], Ashis Borah[a], Takashi Okubo[b#] and Hideto Takami[b$]

[a]Microbiology and Molecular Systematics Lab, Department of Botany, Gauhati University, Guwahati, Assam, India
[b]Yokohama Institute for Earth Sciences, JAMSTEC, Yokohama 236-0001, Japan

*Corresponding author: d_narzary@gauhati.ac.in
[#]Current address: Macrogen Japan Corp., 2-4-32 Aomi, Koto-ku, Tokyo 135-0064, Japan.
[$]Current address: Marine Microbiology, The University of Tokyo, Atmosphere and Ocean Research Institute, 5-1-5 Kashiwanoha, Kashiwa, 277-8564, Chiba, Japan.

Supplementary Data Fig. S2. Biofuel producing enzyme (BPZyme) counts as recorded in the traditional beer starter '*emao*'. BPZymes were annotated after homology search of the non-redundant protein sequences of *emao* against BioFuelDB [49].

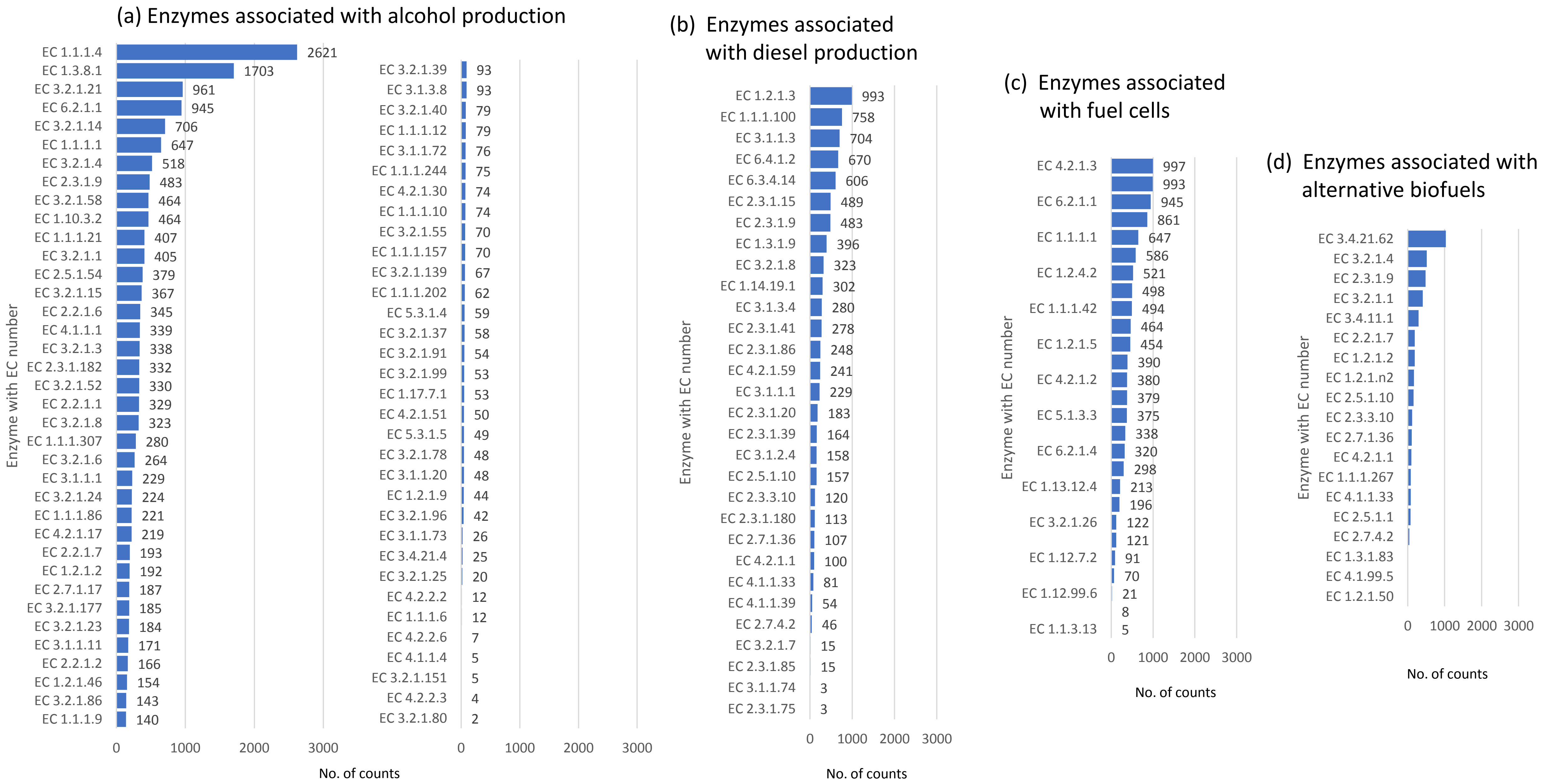

Supplement: Supplementary file 2 — Supplementary Figure S2. [file 41598_2021_94059_MOESM2_ESM.pdf]
