# Supplementary material for: Community structure and metabolic potentials of the traditional rice beer starter ‘emao’
Source: Sci Rep. 2021 Jul 16;11:14628. doi: 10.1038/s41598-021-94059-x (PMC8285430; doi:10.1038/s41598-021-94059-x)
Supplement: Supplementary file 5 — Supplementary Table S3. [file 41598_2021_94059_MOESM5_ESM.docx]

**Community structure and metabolic potentials of the traditional rice beer starter ‘*emao*’**

Diganta Narzary^ab*^, Nitesh Boro^a^, Ashis Borah^a^, Takashi Okubo^b#^ and Hideto Takami^b$^

^a^Microbiology and Molecular Systematics Lab, Department of Botany, Gauhati University, Guwahati, Assam, India

^b^Yokohama Institute for Earth Sciences, JAMSTEC, Yokohama 236-0001, Japan

*Corresponding author: d_narzary@gauhati.ac.in

^#^Current address: Macrogen Japan Corp., 2-4-32 Aomi, Koto-ku, Tokyo 135-0064, Japan.

^$^Current address: Marine Microbiology, The University of Tokyo, Atmosphere and Ocean

Research Institute, 5-1-5 Kashiwanoha, Kashiwa, 277-8564, Chiba, Japan.

**Supplementary Data Table S3.** List of lignocellulose/pentose metabolizing species identified in *emao*. Microbial species recorded in *emao* were cross-verified with secondary sources to identify the activity. RA, relative abundance (in %) of the respective species as recorded in *emao* based on ribosomal protein analysis.

| **Species name** | **%RA** | **Activity** | **Cross Reference (Authors and Journal information)** |
| --- | --- | --- | --- |
| Bacteria |  |  |  |
| *Pediococcus pentosaceus* | 0.30 | Metabolize pentoses (l-arabinose, d-ribose, and d-xylose) | Dobrogosz & DeMoss *J. Bacteriol.* **85(6)**, 1356–1364 (1963). |
| *Leuconostoc gelidum* | 0.11 | Ferments hexoses and pentoses by the phosphoketolase pathway | Jääskeläinen et al. *Appl. Environ. Microbiol.* **81(6)**, 1902–1908 (2015). |
| *Lactococcus lactis* | 0.45 | Assimilate xylose to produce lactic acids | Shinkawa et al. *Appl. Microbiol. Biotechnol.* **91**,1537–1544 (2011). |
| Molds |  |  |  |
| *Mucor circinelloides* | 5.04 | Produces ethanol from D-xylose as well as D-glucose | Takano & Hoshino *Bioresour. Bioprocess.* **5**, 16 (2018).  Komeda et al. *FEMS Microbiol. Lett.* **360**, 51–61 (2014). |
| *Rhizopus delemar*  (syn. *R. oryzae*) | 55.68 | Metabolize xylose and can produce ethanol and lactic acid from rice straw | Maas et al. *Appl. Microbiol. Biotechnol.* **72**, 861–868 (2006).  Abedinifar & Taherzadeh *Biomass and Bioenergy* **33(5)**, 828–833 (2009). |
| *R. stolonifer* | 2.13 | Utilizes substrates containing high cellulose and low lignin, and produces laccase and lignin peroxidase that degrades lignocelluloses | Pothiraj et al. *Mycobiology* **34(4)**, 159–165 (2006).  Kanmani et al. *African J. Biotechnol.* **8(24)**, 6880–6887 (2009). |
| Yeasts |  |  |  |
| *Candida albicans* | 0.14 | Converts starch to ethanol and can grow on xylose medium | Harcus et al. *PLoS ONE* **8(11),** e80733 (2013).  Aruna et al. *Lett. Appl. Microbiol.* **60**, 229–236 (2015). |
| *C. maltosa* | 0.10 | Produce ethanol from xylose and glucose fermentation | Lin et al. *PLoS ONE* **5(7)**, e11752 (2010). |
| *C. orthopsilosis* | 0.08 | Oleaginous yeast that accumulate lipids and can use cellulose as carbon source | Kanti and Sudiana *Curr. Res. Environ. Appl. Mycol.* **5(54)**, 349–56 (2015). |
| *C. tropicalis* | 0.49 | Produce ethanol from xylose | Martins et al. *Brazilian J. Microbiol.* **49**, 162–168 (2018). |
| *C. viswanathii* | 0.08 | Ferments D-xylose to ethanol | Toivola et al. *Appl. Environ. Microbiol.* **47(6)**, 1221–1223 (1984).  Kamble et al. *J. Mol. Catal. B-Enzym.* **35**, 1–6 (2005). |
| *Meyerozyma guilliermondii*  (Syn. *C.* *guilliermondii,* *Pichia guilliermondii*) | 0.06 | Produces ethanol and xylitol from hexoses and pentoses | Saha & Bothast *Appl. Microbiol. Biotechnol.* **45**, 299–306 (1996).  Fonseca et al. *Appl Microbiol Biotechnol.* **75(2)**, 303–310 (2007).  da Cunha-Pereira et al. *Brazilian J. Chem. Eng.* **34(4)**, 927–936 (2017). |
| *Scheffersomyces stipitis* (Syn. *Pichia stipitis*) | 0.07 | Ferments xylose to ethanol | Sanchez et al. *J. Chem. Technol. Biotechnol.* **77**, 641–648 (2002).  Harcus et al. *PLoS ONE* **8(11),** e80733 (2013). |
| *Spathaspora* sp. | 0.05 | Converts D-xylose to ethanol and/or xylitol | Cadete & Rosa *Yeast* **35**, 191–199 (2018). |
| *Ogataea polymorpha* | 0.18 | Ferments xylose and cellobiose to ethanol | Ryabova et al. *FEMS Yeast Res.* **4**, 157–164 (2003).  Kurylenko et al. *Biotechnol. Biofuels* **11,** 197 (2018). |
| *Pichia kudriavzevii* | 0.06 | Ethanol production from acid treated lignocellulose feedstock | Yuan et al. *Microb. Biotechnol.* **10(6)**, 1581–1590 (2017). |
| *Kluyveromyces marxianus* | 0.16 | Produces ethanol from lignocellulose | Goshima et al. *Biosci. Biotechnol. Biochem.* **77(7)**, 1505-1510 (2013).  Sandoval-Nunez et al. *Clean Technol. Environ. Policy* **20**, 1491–1499 (2018). |
| *Pachysolen tannophilus* | 0.56 | Metabolize D-xylose to ethanol | Slininger et al. *Biotechnol. Bioeng.* **24(2)**, 371-384 (1982).  Debus et al. *Eur. J. Appl. Microbiol. Biotechnol.* **17**, 287–291 (1983).  Sanchez et al. *J. Chem. Technol. Biotechnol.* **77**, 641–648 (2002). |
